# Skin prick/puncture testing in North America: a call for standards and consistency

**DOI:** 10.1186/1710-1492-10-44

**Published:** 2014-09-02

**Authors:** Shahnaz Fatteh, Donna J Rekkerth, James A Hadley

**Affiliations:** Allergy, Asthma Care Center of Florida, 33324 Plantation, FL USA; GREER® Laboratories, Inc, 28645 Lenoir, NC USA; Physicians Regional Medical Center, 34119 Naples, FL USA

**Keywords:** Skin testing, Quality, Proficiency, Immunotherapy, Allergen, Extract, SCIT, Disease-modifying

## Abstract

**Background:**

Skin prick/puncture testing (SPT) is widely accepted as a safe, dependable, convenient, and cost-effective procedure to detect allergen-specific IgE sensitivity. It is, however, prone to influence by a variety of factors that may significantly alter test outcomes, affect the accuracy of diagnosis, and the effectiveness of subsequent immunotherapy regimens. Proficiency in SPT administration is a key variable that can be routinely measured and documented to improve the predictive value of allergy skin testing.

**Methods:**

Literature surveys were conducted to determine the adherence to repeated calls for development and implementation of proficiency testing standards in the 1990’s, the mid-2000’s and the 2008 allergy diagnostics practice parameters.

**Results:**

Authors publishing clinical research in peer-reviewed journals and conducting workshops at annual scientific meetings have recommended proficiency testing based primarily on its potential to reduce variability, minimize confounding test results, and promote more effective immunotherapeutic treatments. Very few publications of clinical studies, however, appear to report proficiency testing data for SPT performance. Allergen immunotherapy recommendations are updated periodically by the Joint Task Force on Practice Parameters representing the American Academy of Allergy, Asthma and Immunology (AAAAI), the American College of Allergy, Asthma and Immunology (ACAAI), and the Joint Council of Allergy, Asthma and Immunology (JCAAI).

**Conclusions:**

Despite consensus that all staff who perform SPT should meet basic quality assurance standards that demonstrate their SPT proficiency, the gap between recommendations and daily practice persists. By embracing standards, the accuracy of SPT and allergy diagnosis can be optimized, ultimately benefiting patients with allergic disease.

## Introduction

Skin testing to detect allergen-specific IgE has been in clinical use for over 100 years [1]. Many different techniques and devices have been developed to perform skin tests. Some techniques have been abandoned due to their low reproducibility and painful nature while others have proven useful and continue to be part of the allergy specialist’s practice more than 30 years after their introduction [2]. A skin prick test (SPT) can detect tissue-bound IgE and an atopic state in patients with a type 1 allergy. It can be used to provoke an immediate hypersensitivity response in the skin [3] when the point of the device is used to prick/puncture the stratum corneum, resulting in exposure of the epidermis to an allergen (extract) solution. Antigen presented to tissue mast cells cross-links surface-bound IgE, releasing mediators that stimulate measurable wheal and flare reactions [4].

The prick/puncture method of skin testing is one that has been widely accepted as a safe, dependable, convenient, and cost-effective procedure [3, 5]. Currently, SPT is one of the most widely-used [6] screening [7] and diagnostic tools in modern allergy practice [8] and is considered the “gold standard” method [9, 10] against which other testing methods are sometimes compared. However, it is a method prone to influence by a variety of factors that can add to the overall margin of error, with the potential to significantly alter test outcomes and adversely influence both the accuracy of diagnosis and the effectiveness of the subsequent immunotherapy regimens.

Unintentional variations in SPT [11] techniques can go unnoticed when operator proficiency is not routinely assessed to verify the reliability of- test results. SPT operator proficiency is a variable that is relatively easy to quantify via a coefficient of variation (CV%) in the size of the wheal produced in response to a control or test substance. Because of this simple and readily available measure, routine evaluation of technician technique can be used to help gauge the accuracy of SPT results as part of quality assurance standards.

This study explores the extent to which quality assurance standards related to accuracy/reproducibility testing appear to be both performed and reported in clinical trials and the potential clinical implications of having or not having this information available. This review focused on clinical studies because of the more stringent methodology and accountability associated with clinical trials as compared with the related procedures common in real-world clinical practices. Because recommendations and guidelines for standards of practice are typically evidence-based, (i.e., rely on published clinical trials) it is important that allergy specialists in research and clinical settings acknowledge the importance of an acceptable level of SPT proficiency based on a specific standard. Adopting this practice on a routine basis could possibly bring SPT into better alignment with those areas of medicine where published data require that a standard deviation or standard error is reported, since in the absence of these measures, the data are often considered not meaningful as reported.

## Methods

A predefined literature search strategy was developed to answer the following question: “Of peer-reviewed United States or Canadian publications in which SPT was employed in any part of the study methodology, how many mentioned SPT operator proficiency testing or determination of a coefficient of variation for the operator?” This search was conducted to obtain an appraisal of the transparency associated with reporting tester proficiency in conjunction with the rigorous methodology that is a part of a clinical protocol. The search was limited to the US and Canada because allergic disease is treated very differently in North America and Europe. The search, performed in October 2013 through PubMed and limited to publications within the prior 5 years, used medical subject headings for (“clinical trial” [publication type] or “longitudinal studies”) combined with (“desensitization, immunologic” or “immune tolerance”). The search strategy involved subsequent manual review for inclusion of publications from North American research groups only and exclusion of studies that did not involve immunotherapy, review papers, and animal studies. Allowance was made to consider addition of any other publications of potential relevance. Since this manuscript is a review of a literature search there was no new experimental research carried out on humans or animals.

## Results

A total of 515 citations were generated by the search. Of this number, 471 publications were excluded for the criteria stated above (predominantly the geographic restriction), which yielded 44 publications for review. One additional publication [12] not identified through the search, presumably because it had not been coded as either a “clinical trial” or a “longitudinal study” on PubMed, was added since it met the search parameters. Manual review of the methods sections of these 45 publications to confirm description of skin testing, predominantly as “skin prick testing (SPT)”, lead to the elimination of an additional 5 papers that did not specify that skin testing was performed. Of the remaining 40 publications, only 2 provided any detail about proficiency testing. One paper reported that, “SPTs were performed by S.M.A., who achieved a coefficient of variation of 16.25 on repetitive testing with histamine on a single patient” [13]. The second paper reported that, “An SPT score was computed by subtracting the saline control measure, and a positive SPT response was defined by a score of 3 mm or greater. Tests were considered reliable if the wheal of the negative control (50% glycerin-saline) was 3 mm or smaller and wheal size elicited by the histamine control was at least 3 mm larger than the wheal size elicited by the negative control. All sites used the same lot of reagents, and training was performed to ensure consistency “ [14]. Several other papers also described the methodology for measuring wheal size and comparing with controls, but did not mention methodology for proficiency testing, determination of coefficient of variation, or other measures of quality assurance. This search has many limitations and does not provide an exhaustive survey of the reporting of skin-prick testing in global clinical trials. However, these results lead to the conclusion that the details and reliability of skin-prick testing in clinical studies are rarely described in the current allergy immunotherapy literature. It should also be noted that the failure to report on the proficiency results does not necessarily indicate that it was not performed.

## Discussion

### A brief history of skin testing

Blackley published the results of what is considered by many to be the first skin test in 1873 when he scratched a small area of his own skin, applied grass pollen grains to the abraded area, and noted a large swelling and erythema [8, 11]. Many years later, Schloss introduced the scratch test, a test performed by rubbing an allergen into a small, bloodless abraded area of the forearm [3]. Schick and Cooke independently introduced the intracutaneous test as a diagnostic tool [3]. These early researchers understood the diagnostic value of using a patient’s skin to determine immunologic reactions but refinements were still needed to improve the patient’s experience and the test’s diagnostic potential. Although the scratch test method was used extensively in the past, it became progressively obsolete due to the development of newer, more innocuous procedures with improved accuracy [8]. While many technological improvements have been made in terms of the methods and devices available for allergy testing, the SPT, which is a refinement of the original crude skin test pioneered by Blackley, is still recommended as the primary method for the diagnosis of IgE-mediated allergies in most allergic diseases. SPT was first introduced as a standard test in the 1920s by Lewis and Grant [15] and, following some subsequent modifications [15], remains the standard technique in general use by allergy specialists today [8].

### Options for allergy testing

Skin prick testing (SPT) has become the primary means to confirm an IgE-mediated allergic response [16] because of the numerous advantages it presents to both patients and healthcare providers. It is minimally invasive and allows for the evaluation of multiple allergens in a single session [16, 17]. When performed correctly, skin prick tests are a relatively safe and efficient way to reproducibly diagnose clinical allergy [16–18]. It is a method that is comparatively inexpensive to perform, with results available in 15 to 20 minutes [19], and the test itself evaluates sensitivity based directly on the actual agent of interest [18]. The objective evidence available in the literature supporting the sensitivity, specificity, and positive and negative predictive values of SPT confirms its clinical utility [8].

A variety of *in vitro* serologic test methods can be used to determine serum-specific IgE (sIgE) as an indicator of sensitization to allergens. An *in vitro test*, performed in a clinical laboratory, provides an indication of the level of serum IgE specific to the selected allergens. *In vitro* tests may be the best option for patients with skin conditions that preclude the use of skin testing. It should be noted that laboratories engaged in *in vitro* testing in the United States are regulated by the Clinical Laboratory Improvement Amendments act of 1988 (CLIA), which set standards for testing including training, quality control, and proficiency testing to ensure the reliability, accuracy, and timeliness of patient test results, regardless where the tests were performed [20]. Recommendations for IgE testing from Clinical and Laboratory Standards Institute (CLSI) are that intraassay coefficients of variation on IgE tests not exceed 15% [21].

Sensitization does not always result in clinical consequences. Thus, regardless of the test method chosen, test results must always be interpreted within the context of the patient’s clinical history [3].

Each of these test methods has advantages and disadvantages, summarized in Table 1, that should be considered by the clinician.

**Table 1:** **Comparison of advantages and disadvantages of common allergy test methods**

Type of testing	Test method advantages	Test method disadvantages
SPT	• Minimally invasive	• Can be uncomfortable for some patients
	• Less patient discomfort than ID testing	• Can be contraindicated in patients with extensive skin disease, those taking certain drugs that cannot be discontinued, or those with a recent history of anaphylaxis or current pregnancy
	• Sensitive discrimination between positive and negative results	
	• When properly performed, results are highly specific	
	• Multiple allergens can be tested at one time	
	• Lower rate of systemic effects than intradermal testing	
	• Results available in 15 to 20 minutes	
	• Better correlation with allergy symptoms than in vitro test results	
	• Relatively inexpensive	
Intradermal Testing	• More sensitive than SPT testing	• Is generally less well tolerated than SPT
	• May be more reproducible than SPT testing	• Takes longer to perform than SPT
	• Provides more information on the relative sensitivity of the patient to each allergen tested	• May provide more false positive results than SPT
	• Results available in 15 to 20 minutes	• Requires more technical skill to deliver intradermal injections than SPT
		• Greater risk of systemic reactions than SPT testing & should only be used after a negative SPT result
		• Like SPT, can be contraindicated in patients with extensive skin disease, those taking certain drugs that cannot be discontinued, or those with a recent history of anaphylaxis or current pregnancy
*In vitro* testing	• Single blood draw may be more comfortable for some patients than skin testing	• Results correlate with clinical status less well than *in vivo* test methods
	• Eliminates possibility of systemic reactions	• Results from different methods may not correlate well with each other
	• Can be used on patients who have skin disease that interferes with skin testing	• No standardized reporting of sIgE test results available; this can mask problems with inter-assay variability
	• Can identify sensitivity to cross-reacting allergens	
		• Turn-around time for results longer than skin testing
		• May be more expensive than skin test methods

Despite the fact that its use in some patient populations is problematic (see Table 1), SPT is still considered an effective [22] and useful [23] modality for demonstrating an IgE-mediated underlying mechanism in most suspected allergic disease. Due to its significant role in the diagnosis of allergy and in guiding subsequent immunotherapy, SPT, together with patient history, is typically the preferred first-line diagnostic procedure in working up suspected allergic disease [22].

### Variables that can influence SPT results

Multiple factors can influence the outcomes and interpretation of SPTs. Both controllable and uncontrollable variables can make this seemingly simple, quick, and convenient method for the diagnosis of allergic disease more complex than might initially be appreciated [23–29]. Table 2 categorizes some examples of SPT variables that may or may not be under the control of the test operator. For example, the technician performing the tests cannot influence inherent patient characteristics [29] such as the patient’s age [30, 31], race [8], or any skin damage [16].

**Table 2:** **Variables that can affect SPT results**

Degree of Control Possible	Source of Variability	Variable
Controllable	• Patient Variables	• Choosing the appropriate anatomic site for testing [26–29]
		• Distance between SPT sites [26, 27]
		• Proximity of control tests to the allergen tests [23]
		• Documentation of any unusual skin trauma [16]
	• Performance Variables	• Consistent technique used for administering controls and allergen extracts [23] (e.g., uniformity in the depth of penetration)
Control may depend on who is able to influence certain factors in the clinical/research environment	• Patient Variables	• Awareness of the attenuating/confounding effects of medication [13, 22]
	• Test Supply Variables	• Quality/potency of test allergy extract [22]
		• Source of the extracts [15]
	• Variables in Reading the Test	• Choice of a qualitative, semi-quantitative, or quantitative method for reporting cutaneous reactivity to allergens
Uncontrollable	• Patient Variables	• Age of patient/subject [30, 31, 24]
		• Racial factors (i.e., skin color) [8]
		• Sun damage of skin [13]
		• Existing disease processes, (e.g., hypertension, diabetes, immunodeficiency that may interfere with the development of a skin test reaction) [23]

The reproducibility of SPTs is an indication of how well the controllable variables are being managed. Variability in test results can be largely ascribed to differences in the controllable variables that are tester-dependent; factors such as the angle of the device application, the amount of pressure applied, the distance between SPT sites, and the precision of wheal measurements. The test results will provide accurate and reliable information for clinicians only when deviations due to controllable variables is minimized. Therefore, to optimize the validity of SPT results, it is recommended that technicians who perform SPT undergo regular evaluations of their proficiency in attaining quality assurance standards [16, 32].

### Skin prick testing devices

**Figure 1:**
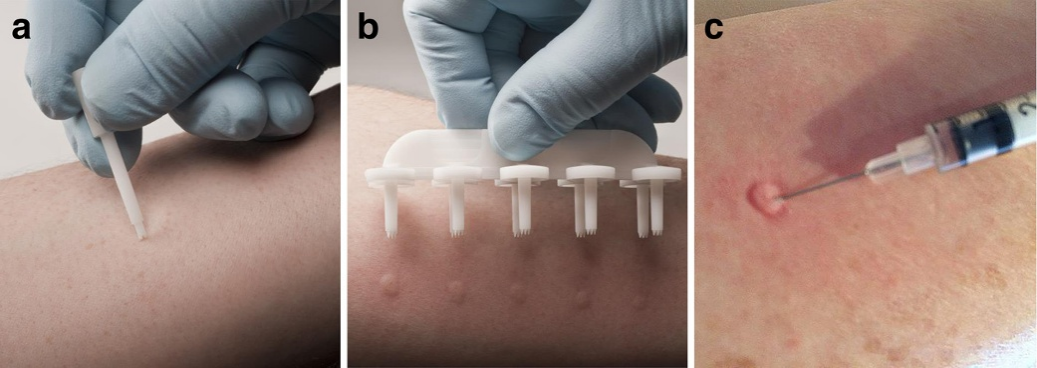
**Examples of single site (a) and multiple site (b) skin prick testing and intradermal testing (c).**

Skin prick testing can be performed with single-site or multiple site test devices (Figure 1). Over past decades, many different skin testing devices have been developed [2], modified, and improved [33–35] with features designed to minimize inaccurate results, lessen patient discomfort [36], streamline test procedures [35], and improve the sensitivity and reproducibility of the results [37]. A wide variety of skin prick test devices are available.

Single-site skin prick devices include steel lancets with surrounding plastic guards to limit penetration depth [29, 38, 39],allergen-coated lancets [40], metallic lancets, and plastic lancets [38]. The size of skin punctures made with these devices is dependent on factors such as needle lengths, point lengths, needle widths, and the pressure and angle of application used by the tester. Different manufactures may also recommend different techniques of application for similarly designed devices [36].

Multiple site test devices streamline testing by allowing the operator to perform up to 10 tests in one application thus both reducing the time required for testing and facilitating practices’ adoption of standard testing panels [41]. Among the many multi-site devices available, there are design differences in the numbers of tines/lancets per stylus, tine/lancet spacing, footprint areas, depth of antigen delivery, and the amount of antigen that is delivered. In multi-site devices, the angle of insertion is fixed, thus reducing the number of variables in testing. However, no device has been shown to completely address all of the controllable variables in skin prick testing [29].

**Figure 2:**
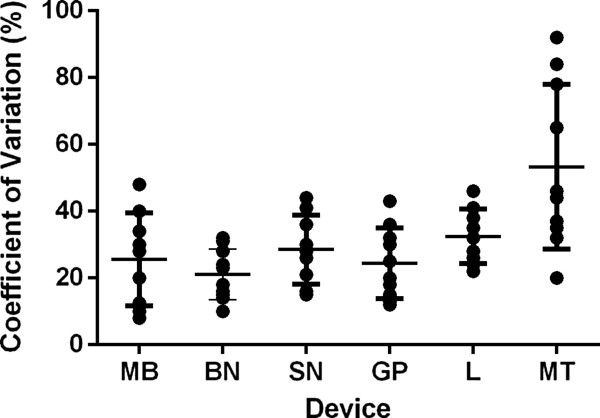
**A comparison of the coefficient of variation (CV% = standard deviation [SD]/mean × 100) for 6 skin test devices is shown for individual subjects** **[**[42]**]** **.** The CVs are for the following devices: Morrow-Brown needle (MB; n = 12), bifurcated needle (BN), SN (smallpox needle; n = 12), GP (GREER “pick”; n = 12), lancet (L), and Multi-Test (MT; n = 15). Each dot may indicate >1 individual and the horizontal bars indicate mean ± SD. MT significantly greater than MB, SN and GP; *p* < 0.05. Adapted from Adinoff, 1989 [42].

In a comparison of six SPT devices tested by Adinoff et al., the mean CV% for the wheal areas produced by skin testing ranged from 22.6% to 39.5%. for the different devices [42]. (Figure 2) Other head-to-head studies have shown that statistically significant differences exist among devices when their performance was assessed by the size of the wheal and flare produced and the sensitivity and specificity of the results after controlling for residual variability or variation between operators and test subjects [36, 43].

Different SPT devices can offer users different potential advantages [4] as well as challenges. ^.^To find the device(s) that best fit a practice requires allergists to carefully evaluate their features [36] and know the limits of the instruments they use [2]. The most recent publication of the updated practice parameters recommends that optimal results can be expected by choosing a single prick/puncture device and properly training skin test technicians in its use [3].

### Importance of SPT proficiency testing

The need for tester proficiency has been demonstrated in a study in which 4 experienced nurses were tasked with performing SPT for grass, dust mite, dog, and mugwort with histamine and saline controls on the same individuals [18]. The target coefficient of variation (CV) between individual nurses was 25% or less [18]. Resulting CVs ranged from 55.9% for saline negative controls to 16.6% for histamine-positive controls. The CVs for assessing dog, grass, dust mite, and mugwort allergens fell in between those values at 43.3%, 42.8%, 26.5%, and 24.7%, respectively. Because of the many additional variables associated with *in vivo* versus *in vitro* test methods, one cannot expect that SPT will have CVs comparable to existing serological methods, but this study illustrates just how variable the performance of SPT can be. This naturally raises the question, “What constitutes an acceptable limit for the accuracy and precision of results related to the performance of SPT?”

It is recognized that the introduction of mandatory proficiency testing can increase the reproducibility of test results [44]. Performance standards and proficiency testing can exert a positive impact on the results obtained and are essential to improving the diagnostic value of clinical allergy skin testing [44].

### Oppenheimer 2006 survey results

In 2006, Oppenheimer and Nelson reported some thought-provoking survey data relating to the extent of diversity in SPT parameters among clinical allergists practicing in the United States [25]. Of the 3000 physicians from the American College of Allergy, Asthma and Immunology (ACAAI) who were invited to respond to a questionnaire, 539 (18%) completed and returned it. Respondents included mostly board-certified allergists (92%) and physicians who had been in practice an average of 7.25 years, with the range for time in practice spanning from 1 to 45 years. Only 1.3% of respondents indicated they did not perform SPT. Among the results reported were the findings that significant variability did exist in a number of skin testing parameters and that quality assurance (reported by a yes/no question on the survey) was performed by 61.2% of the allergists who responded. However, fewer than 10% of those who responded to the survey indicated that they opted to use an objective test protocol (protocols not described) for quality assurance purposes.

The 2006 survey results suggest that more than one-third of skin test technicians may not be routinely assessing their ability to consistently perform SPT and 90% may not be objectively assessing the reproducibility of their technical skills. At least in part, the explanation for this level of indifference to technical performance assessment may be attributed to the ongoing development of SPT devices designed to help reduce the variability of SPT results. Improvements in devices may inspire greater confidence in the potential for operator reliability (e.g., multiple-head SPT devices negate the need to accurately measure the distance between adjacent skin allergen test sites versus single-site allergy skin testing). However, the lack of international agreement [45] and the absence of formal standards for evaluating operator proficiency [25] continue to be key factors contributing to the lack of concern about validating the performance of SPT procedures. Based on these results, it appears that a significant proportion of clinical practices do not routinely verify operator proficiency using objective standards.

### The importance of standards

There have repeatedly been calls for standards for SPT that are evidence-based and reflect best practices. Ideally, such standards would include quality assurance requirements to ensure accuracy of the testing technique and that results are documented in a way that makes them interpretable by other physicians in the event of a second opinion or a patient changing providers [3, 16]. Making the SPT procedure subject to the continuous and strict quality control typical of in vitro diagnostic laboratory methods would achieve this objective [28]. At a minimum, reporting a coefficient of variation for skin tests used in clinical studies and tracking an internal testing standard in clinical practice settings would be positive steps towards maintaining acceptable performance standards [18].

### Proficiency in allergy skin testing: potential standards

Recommendations for proficiency testing standards are evolving. Individual clinicians have offered guidance on research laboratory methods, some of which may serve as a basis for proficiency test standards in clinical settings and others which may be more suitable for research purposes. Addressing the need for proficiency in intradermal testing, Turkeltaub is an advocate of a proficiency test involving testing multiple dilutions of 2 different histamine concentrations on the same subject. When properly performed, the 2 dose–response lines should be parallel. The distance between the two lines defines the relative potency of the two histamine concentrations. Operators are considered proficient in this method if they meet the clinical and statistical criteria for pre-established dose–response lines and if their mean half-maximal dose estimates fall within pre-established confidence limits for accuracy and precision [44]. Another method, recommended by Cox, requires the administration of 10 alternating positive (histamine) with 10 negative (saline) controls. Cox states the quality standard or CV should be less than 30% [16]. Dreborg has stated that skin testing proficiency should be mandatory in scientific trials that include the diagnosis of sensitization or allergy [46]. Dreborg suggests that skin tests should be performed in a standardized manner and held to standards similar to those used to assess “laboratory methods” [47]. These and other clinicians have advocated standardized proficiency testing to minimize the errors that they know from experience can be caused by varying test techniques within and between operators during a clinical trial [46].

A coefficient of variation is a useful statistic for comparing the degree of variation from one series of repeated measurements to another. Because the CV is a normalized standard deviation, it allows comparison of variability estimates regardless of data means. Within-subject SPT proficiency CVs serve as a measure of operator precision and reproducibility. The acceptable CV can vary depending on the method of testing employed. While many laboratory procedures require a CV of less than 5%, this target is generally considered unrealistic for skin tests [47]. For *in vitro* allergy testing, the USA Clinical and Laboratory Standards Institute (formerly National Committee for Clinical Laboratory Standards) recommends quality control procedures, with a target CV of less than or equal to 15% [41]. Data suggest that, for IDST, a CV of less than 10% based on the area of the wheal may be a reasonable standard [47]; however, the CVs for mean wheal diameters in SPT can be above 20% and, for the area of the SPT response, can be more than 40% [47]. CVs to evaluate the performance of the SPT method have been shown to vary markedly between different centers, ranging from less than 15% to in excess of 60% [48] in some instances. Dreborg is in agreement with generally recommended CV values of less than 20% and less than 40% for IDST and SPT, respectively [8, 49]. As early as 1989, the subcommittee on Skin Tests of the European Academy of Allergology and Clinical Immunology provided guidance on the performance precision of SPT for epidemiological studies [50]. They recommended that investigators achieve a CV of less than 40% for an area measurement or less than 20% for a diameter-based measurement based upon a mathematical transformation of the wheal area. This recommendation was based on data showing a CV of 15% and 7%, respectively, which was realistically attainable by highly proficient investigators [50]. The mathematical transformation (^10^log [area mm^2^ + 1]) may, however, not be practical for most clinical practices. A CV of less than 20% for tester proficiency, after repeated histamine control applications, has been recommended in other European publications [31].

In 1993, the Board of Directors of the American Academy of Allergy, Asthma and Immunology (AAAAI) issued a position statement [51] outlining some recommended test performance guidelines including the quality of the allergen extract, age of the patient, seasonal variations, and the importance of avoiding certain drugs and dermographism. However, this group stopped short of endorsing an acceptable standard for performance proficiency [3]. The Childhood Asthma Management Program study published in 2000 stipulated that a CV of less than 30% be achieved to demonstrate proficiency in skin testing [8, 16, 17, 52]. In 2008, the AAAAI and ACAAI jointly issued updated recommended practice parameters [3]. This publication reveals that the AAAAI and ACAAI do recognize that “…considerable care should be given to proper training of skin test technicians” and advocates that skin test performance should be demonstrated by skin testing proficiency protocols to achieve quality assurance among technicians. The proficiency testing and quality assurance standard suggested for a SPT with histamine by the Joint Task Force was a CV less than 30%. In addition, it was recommended that criteria for positive and negative test results should be pre-determined for the specific device in use.

While in the U.S. or Canada there are still no formal criteria required to verify tester proficiency, several publications have suggested best practices [17, 41]. The suggested proficiency testing and quality assurance protocol for skin testing jointly offered by the AAAAI and ACAAI in 2008 Allergy Diagnostic Testing: An Updated Practice Parameter is shown in Table 3 [3].

**Table 3:** **2008 Practice parameter: recommended proficiency testing and quality assurance technique for prick/puncture skin testing** [3]

Procedure	• Using desired skin test device, perform skin testing with positive (histamine 1–10) and negative controls (saline 1–10) in an alternate pattern on a subject’s back
	• Record histamine results at 8 minutes by outlining wheals with a felt tip pen and transferring results with transparent tape to a blank sheet of paper
	• Record saline results at 15 minutes by outlining wheal and flares with a felt tip pen and transferring results with transparent tape to a blank sheet of paper
Calculations	• Calculate the mean and SDs of each mean wheal diameter
	• Determine coefficient of variation (CV) = SD/mean
Quality Standards	• Histamine Control: CV less than 30%
	• Saline Control: All negative controls should be <3 mm wheals and <10 mm flares

Broad implementation of SPT proficiency testing in North America could potentially lead to more consistent testing techniques, reduce high CVs, and provide greater validation of the data and outcomes in both scientific and clinical spheres [46]. Consensus on a testing protocol as well as target levels of reproducibility has been published; implementation of these standards leading to improved care for allergy patients is the next step.

## Conclusions

Allergy SPT is an essential part of the evaluation, diagnosis, and treatment of allergic disease. SPT operator performance is an important variable that can affect the accuracy of SPT diagnostic results and should not be taken for granted. Documenting a test administrator’s proficiency is an indicator of data validity and has been repeatedly called for in the literature. Oppenheimer’s survey results suggested that, in 2006, up to 90% of practicing clinical allergists might not be objectively assessing the reproducibility of their testing staff’s technical skills and suggested a protocol for proficiency testing. The 2008 Diagnostic Practice Parameters recommended proficiency testing and provided a similar protocol. Yet, reports of quality assurance standards achieved for tester proficiency in published clinical studies using the SPT remain conspicuously absent, as demonstrated in the literature search- reported herein. It seems reasonable to question why these practical testing recommendations are not being adopted and to, once again, recommend that all technicians who perform SPT, whether in a research or clinical practice setting, undergo routine evaluation of their proficiency to meet basic quality assurance standards for SPTs, similar to the requirements, mandated by CLIA, for technicians who perform *in vitro* diagnostic testing.. By embracing those standards, the accuracy of this diagnostic tool can be optimized, ultimately improving the diagnosis, treatment, and quality of life for patients with allergic disease.

### Consent

Written informed consent was obtained from the healthy volunteer in Figure 1 for the publication of this report and the accompanying images.
